# *Pax7 *is requisite for maintenance of a subpopulation of superior collicular neurons and shows a diverging expression pattern to *Pax3 *during superior collicular development

**DOI:** 10.1186/1471-213X-8-62

**Published:** 2008-05-30

**Authors:** Jennifer A Thompson, Andreas Zembrzycki, Ahmed Mansouri, Mel Ziman

**Affiliations:** 1 School of Exercise, Biomedical and Health Science, Edith Cowan University, Joondalup Drive, Joondalup, Western Australia 6027, Australia; 2 Max Planck Institute for Biophysical Chemistry, Goettingen, Germany; 3 The Salk Institute for Biological Studies, Molecular Neurobiology Laboratory, La Jolla, CA, USA

## Abstract

**Background:**

*Pax7 *encodes a transcription factor well-established as an important determinant of mesencephalic identity and superior collicular development. *Pax7 *mutant mice, however, present with no obvious morphological impairments to the superior colliculus. This finding is paradoxical and has been attributed to functional redundancy afforded by its paralogue *Pax3*. Here we utilise *Pax7 *mutant mice to investigate the precise role of this important developmental regulator during superior collicular development and neuronal specification/differentiation. We also assess its spatiotemporal relationship with *Pax3 *during embryonic development.

**Results:**

Analysis of the superior colliculus of *Pax7 *mutant and wildtype mice at a variety of developmental timepoints revealed that whilst correct initial specification is maintained, a subpopulation of dorsal mesencephalic neurons is lost at early postnatal stages. Moreover, a comparative analysis of embryonic *Pax3 *and *Pax7 *expression profiles indicate that *Pax3 *expression overlaps extensively with that of *Pax7 *initially, but their expression domains increasingly diverge as development progresses, coinciding spatiotemporally with neuronal differentiation and maturation of the tissue. Furthermore, *Pax3 *expression is perturbed within the CNS of embryonic *Pax7 *mutant mice.

**Conclusion:**

In summary, these results demonstrate that during superior collicular development, *Pax7 *is required to maintain a subpopulation of dorsal, mesencephalic neurons and partially regulates, spatiotemporally, *Pax3 *expression within the CNS. The differential nature of *Pax7 *and *Pax3 *with respect to neuronal differentiation may have implications for future stem cell therapies aimed at exploiting their developmental capabilities.

## Background

It is evident that *Pax7 *is a multiplex contributor to correct CNS development. This is exemplified by dynamic spatiotemporal expression patterns, occurring from early development and persisting in restricted regions throughout adulthood. *Pax7 *expression initially occurs in the neural tube and mesencephalon from very early stages [1,2] and is required for polarisation of the dorsoventral axis of the neural tube [3] and specification of the superior colliculus/tectum from the mesencephalic alar plate [4-7]. In the developing superior colliculus, graded expression of *Pax7 *establishes rostrocaudal and dorsoventral polarity. Expression of *Pax7 *localises within superior collicular neurons as development proceeds. This expression is upregulated during retinal innervation and axonal arborisation but reduced in *Pax6 *(*Sey*) mutant mice [7], with reduced (20–30%) retinal innervation [8], confirming that Pax7-expressing cells are responsive to retinal input. Demonstrated colocalisation in superior collicular neurons with the mapping marker ephrin-A2 validates Pax7 participation in retinotopic mapping [7]. Continued, graded expression into adulthood is thought to maintain a small population of dorsal neurons in the mature colliculus [7,9], although the functional requirement for this feature remains obscure.

Given the aforementioned importance of *Pax7 *in mesencephalic and superior collicular development, the lack of gross defects in this region in *Pax7 *mutant mice is surprising, and points to rescue by the paralogous *Pax3 *gene which has overlapping expression domains [9,10]. Here, within the superior colliculus, we seek to determine the developmental role of *Pax7 *in specification of neurons, and assess its spatiotemporal relationship with *Pax3*. We have analysed *Pax7 *mutant mice [10] relative to wildtype at key stages of development and results indicate that a subpopulation of neurons is lost during early postnatal stages. We show that this apparent loss of neurons is not due to aberrant specification or proliferation, or cell-fate switching/transdifferentiation to the astrocytic lineage, but rather appears due to the inability of *Pax7 *mutant mice to maintain a subpopulation of dorsal superior collicular neurons.

Furthermore, analysis of *Pax3 *expression in embryonic wildtype and *Pax7 *mutant mice indicates crossregulation between paralogues, and illustrates a functional divergence during superior collicular development. We propose that within the superior colliculus initial overlapping *Pax3 *expression ensures correct neuronal specification, and temporospatial separation of expression patterns leads to solitary expression of *Pax7 *during a critical period of neuronal maturation, which abrogates the ability of *Pax3 *to compensate, revealing the aberrant phenotype.

## Results

### *Pax *gene expression patterns

The Pax7 antibody has previously been demonstrated to be suitable for use in mouse tissue by Western Blot analysis [11]. The Pax3 antibody has been tested *in vitro *and *in vivo *for specificity in recognition of mouse Pax3 by Western Blot analysis [12].

### *Pax7 *expression in wildtype mice

**Figure 1:**
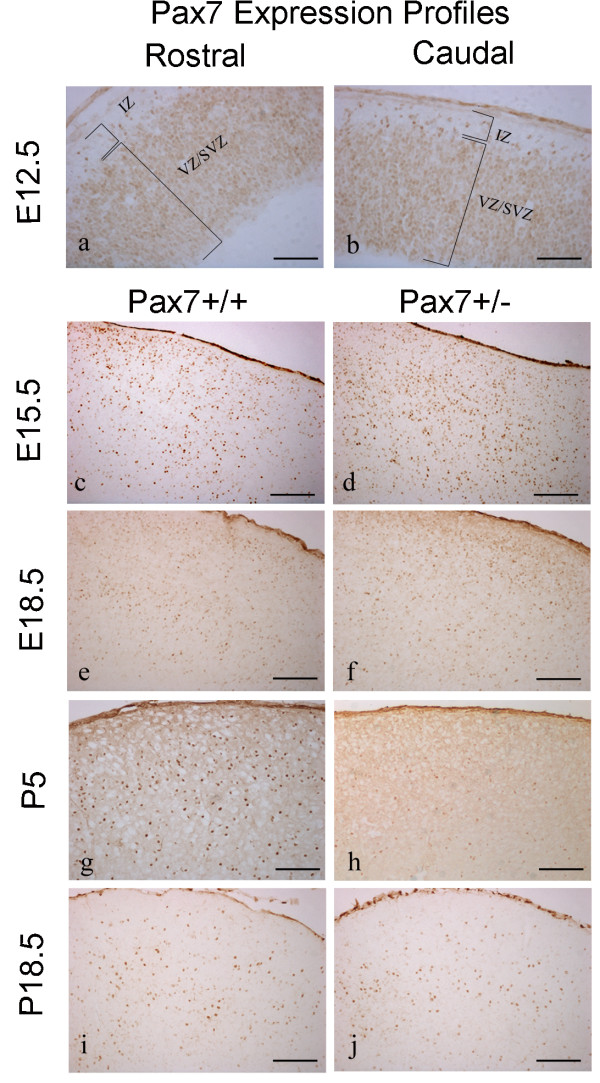
***Pax7 *expression profiles in the developing superior colliculus of *Pax7 *mutant and wildtype mice**. Comparative *Pax7 *expression profiles throughout development of the mouse tectum/superior colliculus in wildtype (a,b,c,e,g,i) and *Pax7*^+/- ^mice (d,f,h,j). Note expression within the intermediate zone (IZ) in both the rostral (a) and caudal (b) regions of wildtype mice at E12.5. The embryonic *Pax7 *profile is comparable between wildtype and *Pax7*^+/- ^mice at E15.5 (c-d) and E18.5 (e-f), however at P5 there is a paucity of Pax7^+ ^cells in the dorsalmost region of *Pax7*^+/- ^mice relative to wildtype (g-h). At P18.5 *Pax7 *expression is similar between wildtype and *Pax7*^+/- ^mice (i-j). Abbrev. *IZ*, intermediate zone; *VZ/SVZ*, ventricular zone/subventricular zone. Scale bar: a-b 100 μm, c-j 200 μm.

**Figure 2:**
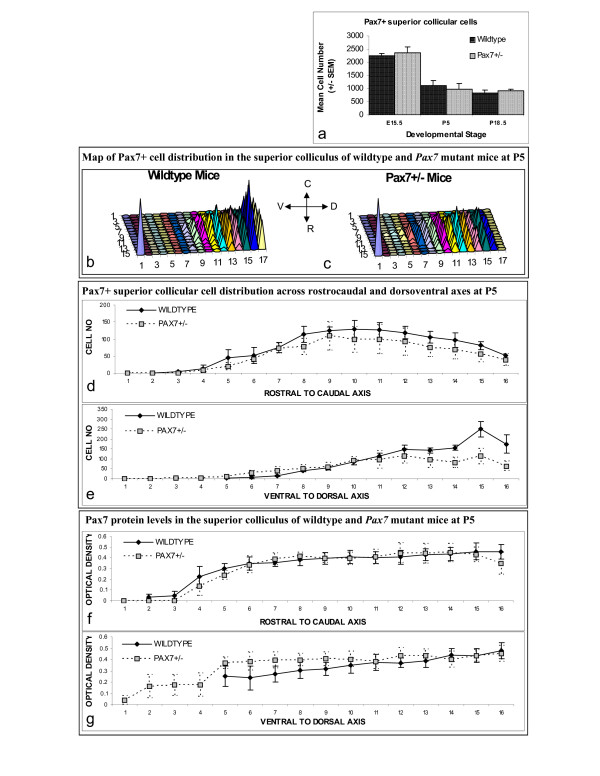
**Quantification of *Pax7 *expression in the superior colliculus of wildtype and *Pax7*^+/- ^mice**. (a) Pax7^+ ^cells in the superior colliculus of wildtype and *Pax7 *mutant mice at E15.5, P5 and P18.5 (n = 3–5). (b-c) Map of Pax7^+ ^cell distribution in the superior colliculus of wildtype (b) and *Pax7*^+/- ^mutant mice (c) at P5 showing reduced Pax7^+ ^cells in the superficial strata of *Pax7*^+/- ^mice. The cone at position 1 represents positive control for relative calibration. (d-e) Mean (+/-SEM) Pax7^+ ^cellular distribution across rostrocaudal (d) and dorsoventral (e) axes. (f-g) Mean (+/-SEM) Pax7 protein levels across rostrocaudal (f) and dorsoventral (g) axes (n = 4 each).

Within the anlage of the tectum, at the earliest embryonic stage examined (E12.5), *Pax7 *expression occurs from the mesencephalic ventricular zone to the most superficial layer. Immunoreactive cells are most dense in the ventricular and subventricular zones, then decrease in the intermediate zone, which is larger rostrally compared to the caudal region at this stage (Fig 1a–b). *Pax7 *expression can also be detected within the subthalamus, pretectum, pons, and in the ventricular zones of the cerebellar primordium, pons and myelencephalon (4^th ^ventricle). This profile concurs with *in situ *hybridisation results reported previously at E13 [9], with the exception of expression detected at the 4^th ^ventricle. At E15.5, a large number of immunoreactive cells are detected (Fig 2a) in the presumptive superior colliculus whilst expression declines in the caudal tectum, reflecting the emerging distinction of the tectum into the inferior and superior colliculi. Pax7^+ ^cells also recede from the mesencephalic ventricular zone, although remnants of expression can still be detected at the dorsal ventricular surface, with cells also noted at the ventral ventricular surface at certain mediolateral positions. Pax7^+ ^cells can be detected up to the pial surface of the superior colliculus with the exception of the stratum zonale, which is now becoming evident (Fig 1c). Expression remains robust within the pons. *Pax7 *expression at E18.5 is similar to that noted at E15.5 (Fig 1e), however immunoreactivity is no longer detected within the ventricular zone and expression within the subthalamus is waning. At P5, while a rostral^low ^to caudal^high^, ventral^low ^to dorsal^high ^gradient is maintained, Pax7^+ ^cell numbers are reduced throughout the superior colliculus (Table 1; Fig 1g; Fig 2a,b,d,e; [7]). Cellular protein levels are similarly graded (Fig 2f–g; [7]). Expression is also reduced at the midbrain/hindbrain boundary, pons and subthalamus. Even fewer Pax7^+ ^cells are detected throughout the young adult superior colliculus (P18.5) (Fig 1i; Fig 2a). Expression persists at the midbrain/hindbrain boundary and rostral to the choroid plexus (4^th ^ventricle), with weak expression in the subthalamus (n = 3–5 at all stages).

**Table 1:** Pax7^+ ^cell distribution in the superior colliculus of wildtype and *Pax7 *mutant mice at P5.

**Pax7^+ ^Cell Distribution**							
**D-V Axis**		**^a^Ventral**	**^a^Mid**	**^a^Dorsal**	**^#^V-M**	**^#^M-D**	**^#^V-D**

**P5**	**+/+**	117.50 ± 8.29	311.50 ± 60.88	551.25 ± 106.79	.051	.025	.025
	**+/-**	126.25 ± 34.97	257.75 ± 56.98	317.25 ± 85.55	.025	>0.05	>0.05

+/+; wildtype +/-; *Pax7*^+/-^

^a ^values are Mean Cell Numbers ± SEM (n = 4).

^# ^indicates a significant p value from paired *t*-test assessing variation between axis points, within wildtype and *Pax7*^+/- ^mice.

### *Pax7 *expression in *Pax7*^+/- ^mutant mice

In *Pax7*^+/- ^embryos, the *Pax7 *expression profile is grossly indistinguishable from that of wildtype mice (Fig 1d,f); cell counts, wt = 2248.67 ± 94.75; *Pax7*^+/- ^= 2373.67 ± 206.88 (p > 0.05; n = 3; Fig 2a). However, at P5, there is a dramatic reduction in the number of Pax7^+ ^cells in the most superficial region of *Pax7*^+/- ^mice relative to those of wildtype mice (Table 1; Fig 1h; Fig 2b–e; n = 4 each). This manifests as a distinct area near the pial surface that is almost devoid of Pax7^+ ^cells uniformly across the entire rostrocaudal (Fig 2d) and mediolateral (data not shown) axes. Moreover, the number of Pax7^+ ^cells is reduced throughout the dorsal half of the superior colliculus. Although the reduction in the total number of Pax7^+ ^cells in *Pax7*^+/- ^relative to wildtype mice at P5 does not reach statistical significance, this phenomenon results in the loss of graded cellular distribution across the dorsoventral axis (Table 1; Fig 2e; p > 0.05). Concomitantly there is some variation in cellular protein levels ventrally creating a lack of graded expression medially (Fig 2g). Interestingly, at P18.5 we did not discern any differences in *Pax7 *expression between heterozygous and wildtype mice; cell numbers, wt = 828.5 ± 108.97; *Pax7*^+/- ^= 913.60 ± 69.77 (p > 0.05, Table 1; Fig 1i–j; Fig 2a; n = 3–5 at all stages).

### *Pax3 *expression in wildtype and *Pax7 *mutant mice

**Figure 3:**
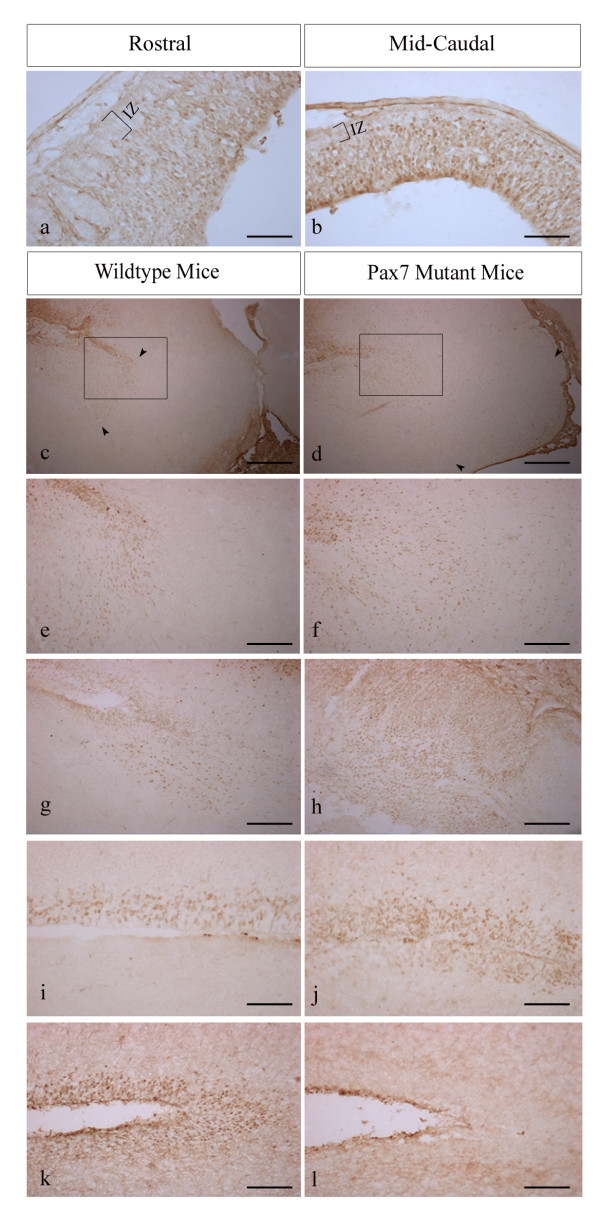
***Pax3 *expression profiles in the embryonic superior colliculus of *Pax7 *mutant and wildtype mice**. Comparative *Pax3 *embryonic expression profiles in wildtype (a,b,c,e,g,i,k) and *Pax7 *mutant mice (d,f,h,j,l). Note the decreased intensity of Pax3 in the rostral region (a) relative to the caudal region (b) of the ventricular/subventricular zones at E12.5, and lack of immunoreactivity within the intermediate zone. At E15.5, Pax3^+ ^cells are present in the hindbrain in wildtype mice (c, e [inset from c]) but are spatially expanded in *Pax7 *mutant mice (d,f [inset from d]). (Arrows denote spatial extent of expression). At the rostral mesencephalon at this timepoint there are increased numbers of Pax3^+ ^cells in *Pax7*^-/- ^mice relative to wildtype (g-h) together with expanded expression at the ventral ventricular surface (j), whereas Pax3 immunoreactivity was only detected in the dorsal ventricular region in wildtype mice (i). Consequently, at E18.5, whilst Pax3^+ ^cells can be clearly detected in the ventricular zone of wildtype mice (k) they are no longer present in *Pax7*^-/- ^mice (l). Abbrev. *IZ*, intermediate zone; *VZ/SVZ*, ventricular zone/subventricular zone. Scale bar: a-b, i-l 100 μm; c-d 500 μm; e-h 200 μm.

At E12.5, in wildtype and *Pax7 *mutant mice *Pax3 *expression can be detected from the pretectum to the tectum in the ventricular and subventricular zones, with rostral expression lower in intensity relative to the mid-caudal region (Fig 3a–b). Immunoreactivity is also evident in the ventricular zones of the cerebellar primordium, pons and myelencephalon, and at the midbrain/hindbrain boundary. By E15.5 *Pax3 *expression within the superior colliculus of wildtype mice is primarily restricted to the dorsal ventricular zone, and the pons (Figs 3c,e). In *Pax7*^+/- ^mutant mice at this stage, *Pax3*^+ ^expression expands ventrally within the myelencephalon and pons (Figs 3d,f). In striking contrast to wildtype (Fig 3g) and *Pax7*^+/- ^mice, *Pax7*^-/- ^mice demonstrate large numbers of Pax3^+ ^cells above the rostral ventricular zone (3^rd ^ventricle) (Fig 3h). Furthermore, Pax3^+ ^cells can be detected at the ventral ventricular surface in *Pax7 *mutant mice but not in wildtype mice (Figs 3i–j). At E18.5, in wildtype (Fig 3k) and heterozygous mice the pretectum and ventricular zone of the superior colliculus remain Pax3^+^, although only a remnant of expression within a small number of weakly stained cells remains within the superior colliculus and in the pons. By contrast, Pax3 immunoreactivity cannot be detected within the ventricular zone of *Pax7*^-/- ^mice (Fig 3l) (n = 2–4 at all embryonic stages). *Pax3 *expression could not be detected at P5 in wildtype or mutant mice (data not shown).

### Spatiotemporal assessment of neuronal proliferation and differentiation within the superior colliculus

To assess whether the loss of Pax7^+ ^cells in the dorsal superior collicular region of *Pax7 *mutant mice was due to altered proliferation we explored *Ki67 *expression at E12.5. We could not detect any variation in expression patterns between wildtype and mutant mice (data not shown), indicating that cellular proliferation proceeds normally, thus excluding neuronal precocity at this stage (n = 3–4).

We next sought to relate observed differences in the *Pax7 *and *Pax3 *expression profiles to temporal differences in neuronal differentiation. We have shown previously that Pax7 co-localises with βIII-tubulin and ephrin-A2 in the mouse superior colliculus at P5 [7]. We therefore used these and other markers of early neuronal differentiation, α-internexin and doublecortin as well as the mature neuronal marker, NeuN to analyse neuronal differentiation in wildtype and mutant mice.

**Figure 4:**
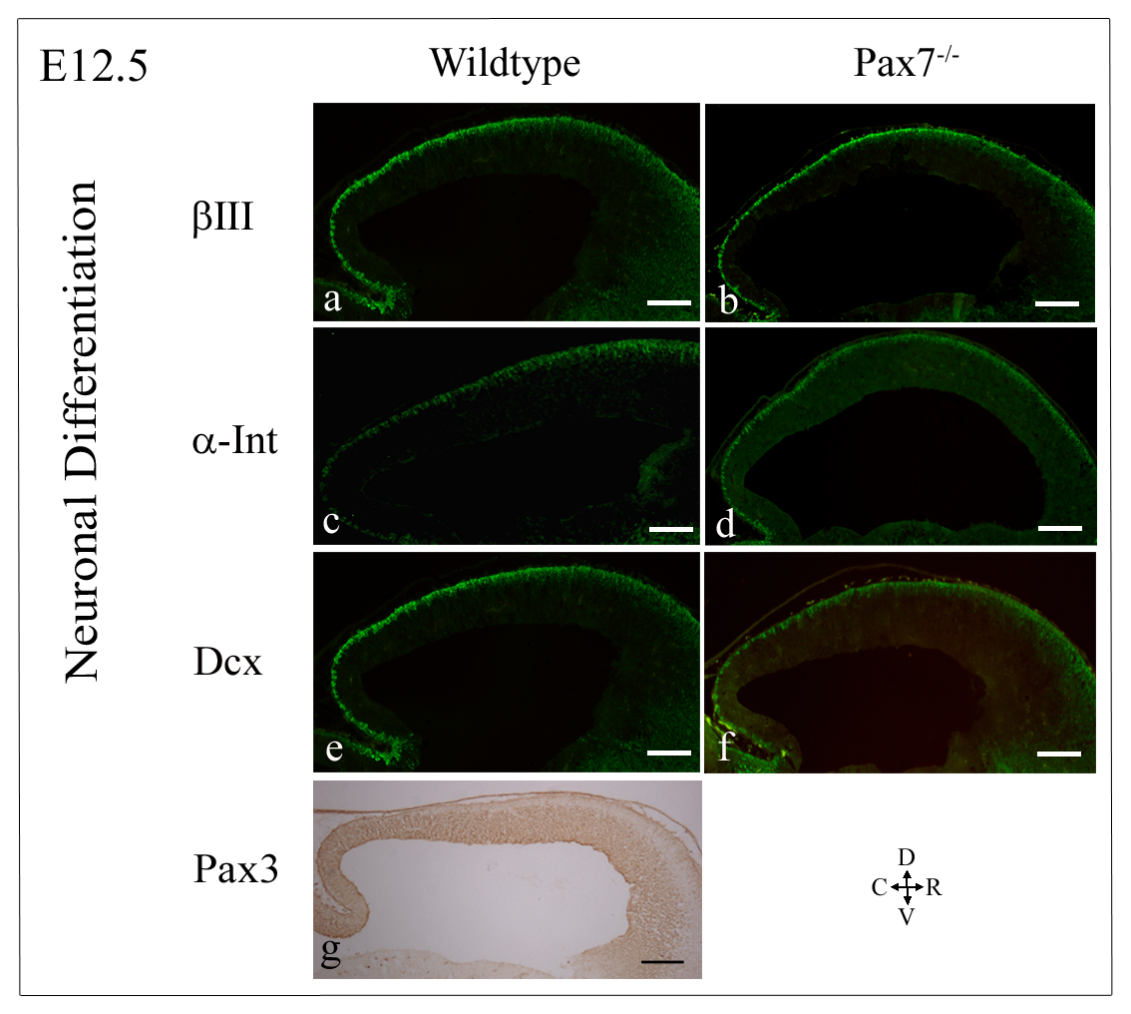
**Comparative neuronal differentiation in the developing superior colliculus of *Pax7 *mutant and wildtype mice at E12.5**. Neuronal differentiation in the developing mouse tectum/superior colliculus of wildtype (a,c,e) and *Pax7*^-/- ^mice (b,d,f) at E12.5, as indicated by βIII-tubulin (a-b),α-internexin (c-d) and doublecortin (e-f) immunostaining. Comparison shows similar neuronal differentiation, with increased spatial expression rostrally relative to caudally, with the rostral region slightly variable at different positions across the mediolateral axis, and neuronal differentiation mid-caudally restricted to the intermediate zone at the pial surface. Compare to the complementary expression of *Pax3 *at E12.5 (g). Scale bar: 200 μm.

The efficacy of βIII-tubulin to discriminate differentiating neurons and tract formation in the mouse embryo has been demonstrated previously [13]. At E12.5 we observed βIII-tubulin^+ ^cells predominantly located within the intermediate zone at the pial surface of the tectum, identifying the emerging *stratum profundum *(SP) (Fig 4a) containing the first differentiating neurons of the superior colliculus [14]. Immunolabelled cells were also observed further ventrally at the rostral tectum, but were reduced in number throughout the mid-caudal region, indicating a rostral to caudal cellular maturation, with the occasional immunoreactive cell seen in more ventral regions.

At E15.5 the SP is clearly visible in the ventral tectum, and immunolabelled, horizontally-oriented processes are now visible extending throughout the dorsorostral superior colliculus (see Additional file 1). These processes extend more caudally at E18.5 and most likely represent the incoming axons of retinal ganglion cells, the major afferent projection to the superficial superior colliculus [15].

When we compared *βIII-tubulin *expression in the embryonic superior colliculus of wildtype mice to that of *Pax7 *mutant mice we could not discern any differences in expression profiles (Fig 4a–b) (n = 2–3 at all embryonic stages).

Similar results were obtained at E12.5 for α-internexin (Fig 4c–d; n = 3), and doublecortin (Fig 4e–f; n = 3), early markers of postmitotic [16] and migrating and differentiating [17,18] neurons, respectively. Results confirm that in mutant mice (n = 3 each) at E12.5, neuronal differentiation occurs normally within the intermediate zone close to the pial surface in a rostral^high ^to mid-caudal^low ^manner.

**Figure 5:**
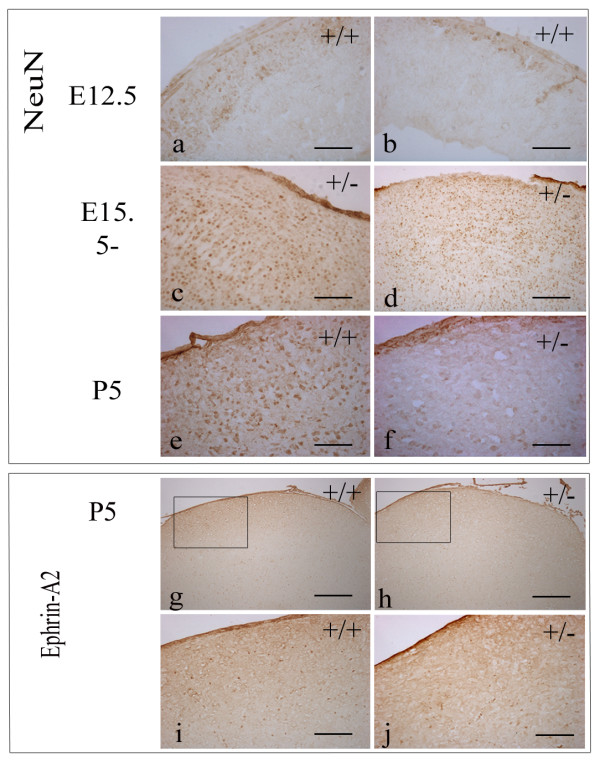
**Comparative *NeuN *and *ephrin-A2 *expression profiles in the superior colliculus of *Pax7 *mutant and wildtype mice**. *NeuN *expression demonstrates increased rostral expression (a) compared to caudal expression (b) at E12.5, indicating that rostral maturation precedes that of the caudal region. Figures (c) and (d) illustrate a full complement of NeuN^+ ^cells dorsally at E15.5 and E18.5, respectively, in *Pax7 *mutant mice consistent with wildtype expression (data not shown). Comparison at P5 between wildtype (e) and *Pax7 *mutant mice (f) indicates a loss of neurons dorsally. Likewise, analysis of *ephrin-A2 *expression shows perturbation between wildtype (g,i) and *Pax7 *mutant mice (h,j) in the dorsalmost region. Scale bar: a-c,e-f 100 μm; d,i-j 200 μm; g-h 500 μm.

During all embryonic stages investigated, we could not detect any difference in the cellular distribution of NeuN^+ ^within the superior colliculus of *Pax7 *mutant mice relative to that of wildtype mice (n = 2–3). The tectum of wildtype mice at E12.5 contains a small number of postmitotic, NeuN^+ ^cells within the intermediate zone, distributed in a rostral^high ^to caudal^low ^manner (Fig 5a–b). At all other embryonic stages examined, NeuN^+ ^cells are distributed throughout the superior colliculus from ventral to pial surfaces (Fig 5c–d).

By contrast, at P5, the most superficial region of the superior colliculus of *Pax7 *mutant mice is almost completely devoid of NeuN^+ ^cells (Fig 5f) indicating dorsal neuronal loss at this important developmental time point. NeuN^+ ^cells can be detected throughout the superior colliculus and are in close proximity to the pial surface in wildtype mice (Fig 5e) (n = 3–6). Interestingly, within the adult mesencephalon (P18.5) *NeuN*, like *Pax7*, shows similar expression in both mutant mice and wildtype mice (n = 2–5).

### Alterations to superior collicular polarity in *Pax7 *mutant mice

Loss of Pax7^+ ^cells and neurons in the dorsal superior colliculus at P5 (Fig 1g–h; Fig 2a–e; Fig 5e–f), when retinal ganglion cell axonal innervation and arborisation is occurring [15], would be expected to impact on superior collicular polarity and retinotopic mapping. We therefore assessed *ephrin-A2 *expression in *Pax7 *mutant mice relative to expression in wildtype littermates. Consistent with the altered *Pax7 *and *NeuN *profile, at P5 ephrin-A2^+ ^cells are missing in the most superficial region (Fig 5h,j), in contrast to wildtype expression displaying immunoreactivity close to the pial surface (Fig 5g,i), providing further evidence of neuronal loss and changes to polarity.

### Neuronal loss is not due to astrocytic cell-fate switching or transdifferentiation

**Figure 6:**
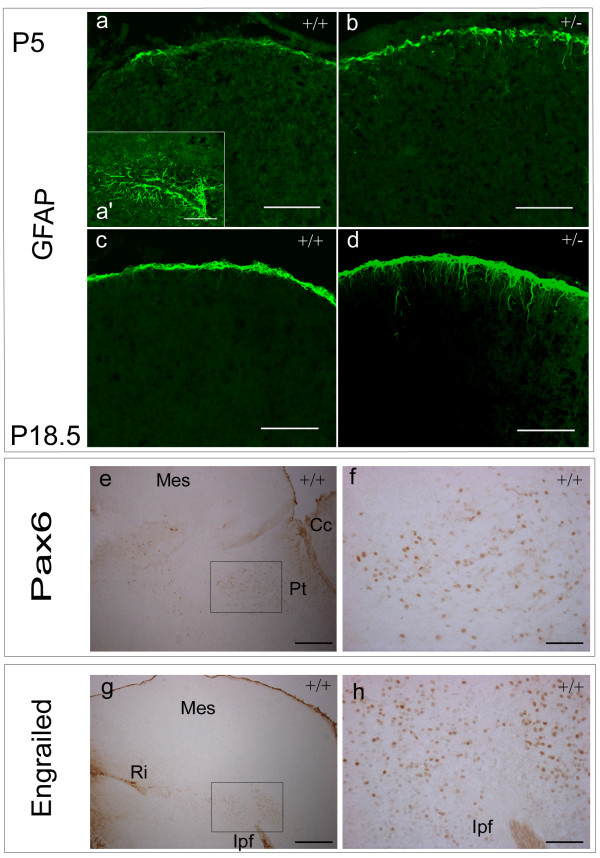
**Comparable astrocytic profile in the dorsal superior colliculus of wildtype and *Pax7 *mutant mice**. Immunofluorescent detection of GFAP indicates a normal astrocytic profile at P5 (a-b) and P18.5 (c-d) for wildtype (a,c) and *Pax7 *mutant mice (b,d). (a') Positive control indicating *GFAP *expression in astrocytes of the myelencephalon. GFAP+ processes extend from cells located at the pial surface, however the dorsal half of the superior colliculus in *Pax7*^-/- ^mice, like that of wildtype, is not populated by GFAP+ cells. Therefore, cell fate switching and/or transdifferentiation towards the astrocytic lineage does not account for the reduction in Pax7^+ ^cells dorsally. Immunohistochemical detection of Pax6 (e, f, [inset from e]) and Engrailed (En-1) (g, h [inset from g]) was utilised to examine mesencephalic boundary formation, which appear morphologically unaffected in *Pax7 *mutant mice. Abbrev. *Cc*, cerebral cortex; *Ipf*, interpeduncular fossa;*Mes*, mesencephalon; *Pt*, pretectum; *Ri*, rhombencephalic isthmus. Scale bar: a-d,f,h 100 μm; e,g 500 μm.

To assess the likelihood of astrocytic cell-fate switching or transdifferentiation, we analysed GFAP expression in postnatal stages as the majority of gliogenesis occurs within the first postnatal week [19]. We could not detect co-expression of *Pax7 *and *GFAP *at P5 or P18.5 in either wildtype or *Pax7 *mutant mice, consistent with previous results determined in the chick tectum [20]. GFAP^+ ^processes can be seen extending from cells located at the pial surface, however the dorsal regions of the superior colliculus in *Pax7 *mutant mice are similar to wildtype mice and are not populated by GFAP^+ ^cells within the region of perturbation at P5 or P18.5 (Fig 6a–d), indicating that cell fate switching or transdifferentiation to the astrocytic lineage is not responsible for the reduced dorsal expression of *Pax7*, *NeuN *and *ephrin-A2 *at this stage (n = 3–4).

### Neuronal loss may be attributable to cellular regression

To evaluate whether neuronal loss was due to apoptosis, we investigated activated *caspase 3 *expression from E15.5 to P5, however we did not detect either co-expression with *Pax7 *at any stage, or increased numbers of cells in mutant mice relative to wildtype (data not shown). This marker, however, proved difficult to analyse as elevated and widespread immunoreactivity detected at the earlier embryonic stages tested may be due to the emerging role of *caspase 3 *in neuronal differentiation [21]. We therefore utilised Hoechst as an indicator of pyknotic nuclei, and assessed co-expression with activated *caspase 3 *as confirmation of the apoptotic status of the cells, but only a few apoptotic cells were detected at any given time point. The identification of apoptotic cells would, however, require knowledge of the exact timeframe in which the neurons were lost, due to rapid neuronal degeneration noted previously [22].

### Mesencephalic boundary formation

To explore the effects of perturbed *Pax7 *expression on formation of superior collicular boundaries, ie between the diencephalon and mesencephalon and between the superior colliculus (dorsal) and tegmentum (ventral mesencephalon), we assessed *Pax6 *expression at the rostral and ventral boundaries of the superior colliculus [4,9,23]. At E12.5, there are a few weakly-stained Pax6^+ ^cells superior to the interpeduncular fossa. At E15.5 in wildtype mice, Pax6^+ ^cells can be detected dorsally in the pretectum (Fig 6e,f) sometimes spreading to the rostral margin of the ventricular zone. Pax6 immunoreactivity could not be detected within the pretectum at E18.5 in either wildtype or mutant mice. We could not detect any abnormality in the expression domain of *Pax6 *in *Pax7 *mutant mice at these midgestational stages (n = 3).

To investigate alterations to mesencephalic/metencephalic boundary formation, we assessed the expression of the marker *En-1 *[24] from E15.5 to P18.5. En-1^+ ^cells can be detected at the midbrain/hindbrain boundary, from the interpeduncular fossa to the rhombencephalic isthmus (Fig 6g,h) and expression wanes as development proceeds until P18.5, when *En-1 *expression in the midbrain is usually restricted to a cluster of cells superior to the interpeduncular fossa. Throughout all stages examined, we could not detect any difference between wildtype and mutant mice (n = 2–3).

## Discussion

In order to understand the role of *Pax7 *in superior collicular development we investigated the superior colliculus of *Pax7 *mutant mice relative to wildtype at a variety of developmental stages. Whilst both *Pax3 *and *Pax7 *are known to define the early tectum subsequent to mesencephalic determination, and the ability to form ectopic tectum subsequent to misexpression within the diencephalon and ventral mesencephalon testifies to their critical nature in specification of tectal identity [5], individual roles during further development have remained elusive. Here we show that loss of Pax7 dramatically alters *Pax3 *expression domains and disturbs the neuronal profile in the dorsal layers of the developing superior colliculus.

### *Pax7 *and *Pax3 *have separate roles during neuronal differentiation in the superior colliculus

Since *Pax7 *and *Pax3 *have similar expression domains in the developing superior colliculus at mid-gestation [9], we undertook a thorough, comparative analysis of their expression patterns in wildtype and mutant mice. It was anticipated that this analysis would highlight any changes caused by loss of Pax7 and shed light on their individual roles during superior collicular development.

**Figure 7:**
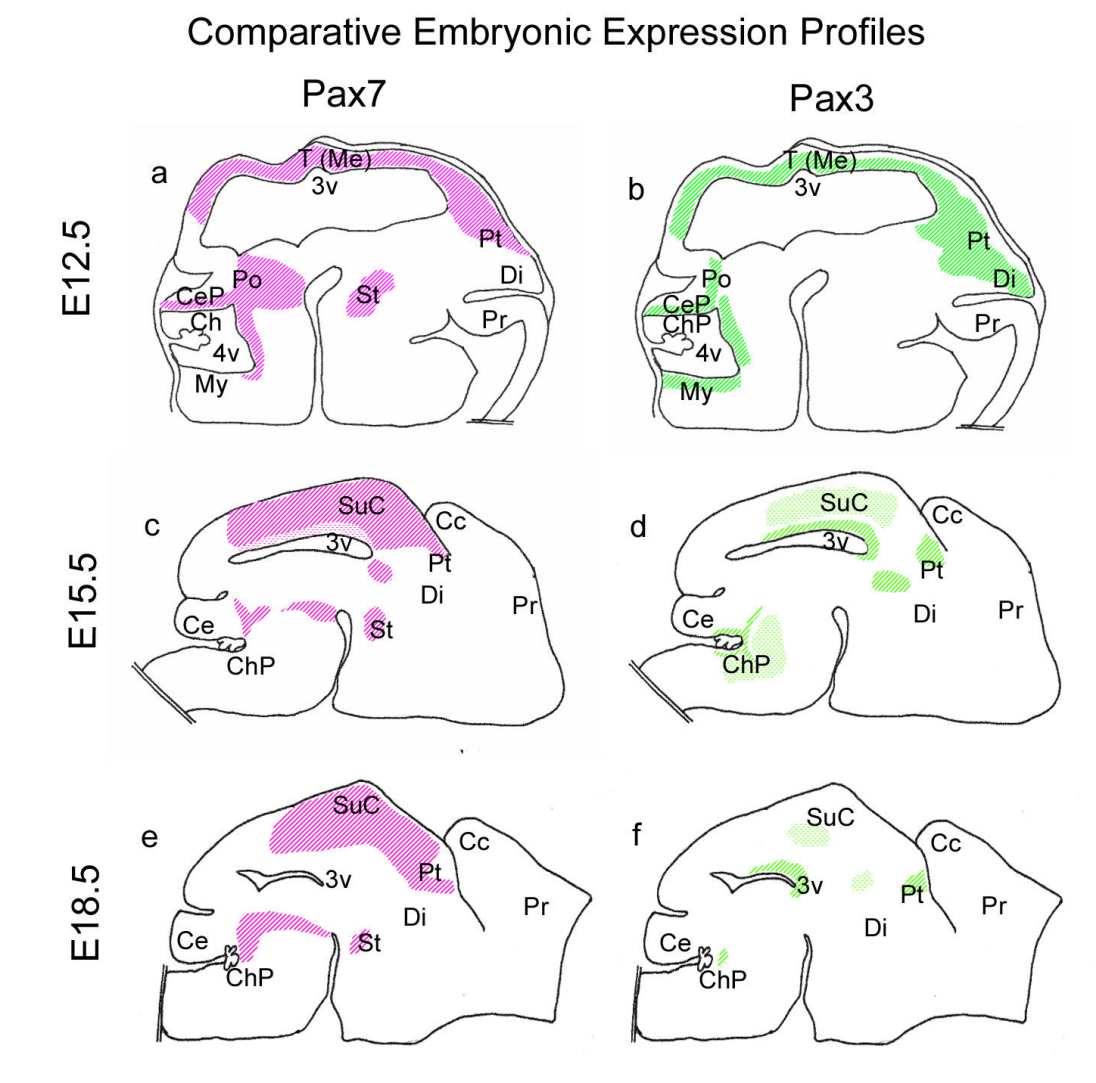
**Schematic illustration of divergent *Pax7 *and *Pax3 *expression profiles in the embryonic mouse superior colliculus**. Divergent *Pax7 *(a,c,e) and *Pax3 *(b,d,f) embryonic expression profiles in wildtype mice at E12.5 (a-b), E15.5 (c-d) and E18.5 (e-f). The lighter pattern for *Pax7 *and *Pax3 *indicates low levels of expression compared to other regions (such as developing upper strata or ventricular zone, respectively) showing darker staining. Abbrev. *Cc*, cerebral cortex; *Ce*, cerebellum; *CeP*, cerebellar primordium; *ChP*, choroid plexus; *Di*, diencephalon; *My*, myelencephalon; *Po*, pons; *Pr*, prosencephalon; *Pt*, pretectum; *St*, subthalamus; *SuC*, superior colliculus; *T (Me)*, tectum (mesencephalon); *3v*, 3^rd ^ventricle; *4v*, 4^th ^ventricle. Illustrations are not to scale.

Results indicate that as the superior colliculus emerges and the dorsal laminae are formed by cellular migration and differentiation, *Pax7 *expression becomes progressively more refined towards the upper, developing strata whereas *Pax3 *expression becomes increasingly restricted towards the proliferative region (Fig 7). At E12.5, *Pax7*- and *Pax3*-expressing cells overlap in the ventricular and subventricular zones, but not in the emerging, differentiating intermediate zone.

At E15.5 tectal *Pax7 *expression within the ventricular zone decreases, and is no longer evident at E18.5, with expression persisting within the upper developing primary layers (*strata profundum, intermedium and superficiale*). However, *Pax3 *expression becomes restricted to the ventricular zone, with a few scattered cells within the intermediate region at E15.5 decreasing at E18.5. Postnatally, expression of *Pax7 *is detected throughout the superior colliculus, numerically declining with maturation yet persisting at all developmental stages examined, whilst *Pax3 *expression is extinguished.

The divergent *Pax3/Pax7 *expression profiles, as demonstrated in this paper, suggest a dichotomy of function with respect to neuronal differentiation. The *Pax7 *expression profile, without co-expression of *Pax3*, appears spatially associated with neuronal differentiation and maturation of the superior colliculus, whilst *Pax3 *expression appears to be negatively associated with these characteristics. A role for *Pax3 *in maintaining the undifferentiated phenotype has been demonstrated previously, both *in vitro *and *in vivo *for neuronal cells [25,26], for Schwann cells [27] and for melanoblasts [28,29]. Conversely, transfection of *Pax7 *into P19 mouse embryonal cells *in vitro *directs these cells along a neuronal pathway [30]. This dichotomy may, therefore, represent an important functional divergence between paralogues which may have implications for future stem cell therapies designed to treat midbrain disorders.

To investigate this further, we performed a comparative assessment of *βIII-tubulin, α-internexin, doublecortin *and *NeuN *expression at E12.5, just prior to the normal birthdate of the superficially located neurons. At this timepoint, the intermediate zone appears as an expanded region rostrally (which varies slightly at different positions across the mediolateral axis) diminishing to a thin region at the pial surface mid-caudally, indicating a rostral to caudal maturity, and is characterised by the expression of *Pax7*, *βIII-tubulin *(somatic staining), *α-internexin, doublecortin *and *NeuN*, and morphologically by increased internuclear distances. By contrast, *Pax3 *expression cannot be detected within the intermediate zone. We could not discern any evidence of precocious neuronogenesis at this time, a function previously demonstrated for *Pax6 *within the eye of *Sey *mutant mice [31]. However, a more thorough investigation at closer temporal increments from E13 would be required to thoroughly preclude this possibility, as precocious or more rapid neuronogenesis may occur between the time frames investigated in this study. Furthermore, the perturbation to *Pax3 *expression within the ventricular zone at the later embryonic stages investigated, suggests that the early processes of specification and/or migration may be accelerated somewhat with insufficient Pax7 levels. The unaltered proliferation/specification of tectal cells does not preclude a role for *Pax7 *in these processes *per se*, but may indicate compensation via *Pax3*.

The observed perturbations in *Pax3 *expression in *Pax7 *mutant mice may indicate that *Pax7 *acts to limit the expression domain of *Pax3*. This is supported by the rostral, ventral and caudal expansion of Pax3^+ ^cells in the presence of reduced or absent *Pax7 *expression. These regions of expanded expression occur where *Pax7 *expression would normally be encountered, and suggests that *Pax7 *acts to dorsalise (or at least positionally constrain) *Pax3 *expression. A cell autonomous relationship between *Pax7 *and *Pax3 *has been determined whereby misexpression of one paralogue represses expression of the other, and a balancing mechanism may exist to produce correct total expression levels [5]. The exact mechanism behind this relationship is ambiguous, however it may be significant that the expression of *Pax7 *precedes that of *Pax3 *within the headfold and primitive fold ectoderm in the developing chick [2]. Moreover, it is clear that this relationship is also dosage-sensitive, as perturbations occur in the haploinsufficient state.

### Role for *Pax7 *in timing of neuronal specification

Expression analysis and cellular quantification have demonstrated that *Pax7 *haploinsufficiency does not alter the initial proliferation steps in Pax7^+ ^cells during mid embryonic stages. The only notable disparity to *Pax7 *expression occurs at P5, where a subpopulation of Pax7^+ ^cells, situated in the dorsal half of the superior colliculus, is no longer detectable, presenting with an obvious margin lacking Pax7^+ ^cells superficially. Sagittal and coronal sections have identified that this perturbation occurs across the dorsal surface of the entire rostrocaudal axis and most of the mediolateral axis.

**Figure 8:**
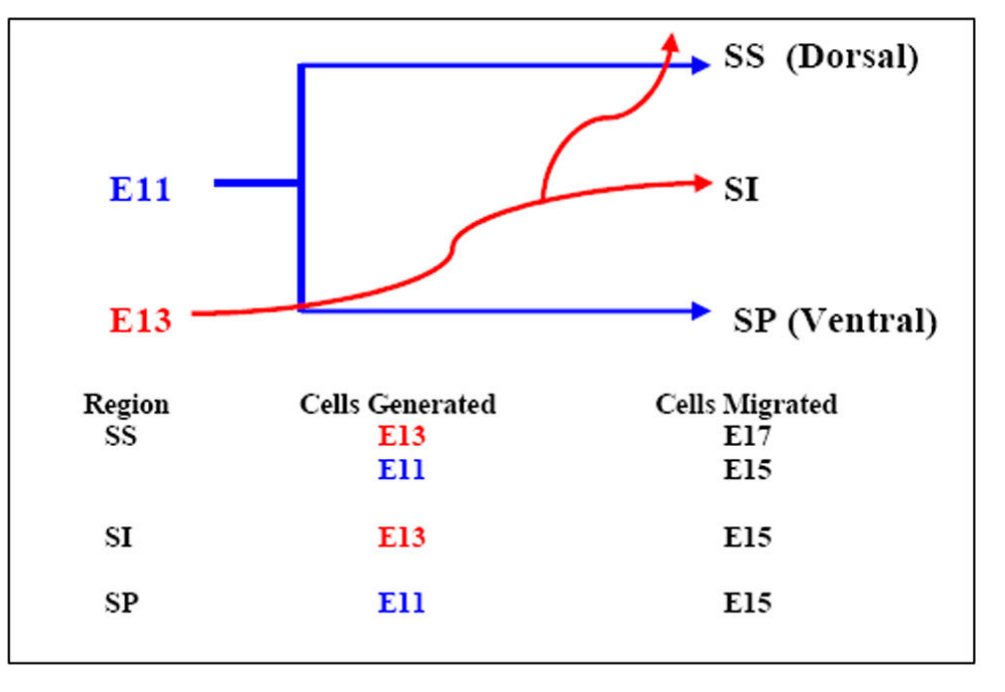
**A diagrammatic representation of the primary layers of the developing superior colliculus**. The laminae of the developing superior colliculus contain two populations of cells that populate the upper (SS), mid (SI) and lower (SP) regions. At E15, cells generated at E11 populate the SS and SP, whereas cells generated at E13 intersect these regions to populate the SI, with a subsequent migration of a subpopulation upwards to the SS by E17. Thus, the SS consists of two different populations of cells, with the most recently generated cells residing in the more superficial regions. Adapted from [14]. Abbrev. *SS*, strata *superficiale*; *SI*, *intermedium*; *SP*, *profundum*. "Cells Migrated" column relates to the developmental timepoint when the cells have migrated to their final destination.

To understand the significance of this anomaly we reviewed previous studies, which indicate that the majority of superior collicular neurons are born throughout the period E11 to E13 [14,19] and distinct temporal and spatial migration patterns culminate in dictation of the correct cytoarchitecture of the developing superior colliculus (Fig 8). Cells generated at E11 initially migrate to populate the superior colliculus, and are subsequently divided dorsoventrally by cells generated at E13 [14]. The cells that eventually reside within the most superficial region of the superior colliculus undergo their final postmitotic division at E13 while in the ventricular/subventricular zones, and migrate to subdivide their predecessors in the intermediate zone at E15. Thereafter, a subpopulation assume their final position superficially at E17 [14] which coincides with retinal ganglion cell innervation of both superficial and intermediate zones [15,32]. The *stratum superficiale *thus contains cells generated at both E11 and E13, with the latter cells being more superficially placed. In contrast, the ventral regions of the superior colliculus, associated with auditory and motor responses, undergo earlier development than the superficial regions and are thus populated by earlier-born neurons [14,33,34]. This temporal specification of cells is similarly observed in the chick tectum, where two populations of cells are generated, each with distinct laminar fates, with later born neurons expressing *Pax7 *fated to reside in the more dorsal laminae [35]. It is therefore evident that the timely migration of cells from the ventricular zone contributes to the correct placement of cells within the superficial laminae, and this temporal-based specification of neurons ensures that placement of cells within laminae coincides with their maturation and initiation of circuitry.

Overall, our results tend to suggest that *Pax7 *may partially contribute to the correct timing of neuronogenesis by regulating migration of Pax3^+ ^cells from the ventricular zone into the mid-collicular region in a timely and orderly fashion, a characteristic that is lost in *Pax7*^-/- ^mice between E15.5 and E18.5, at the time when wildtype cells destined to reside in the more superficial strata are migrating and assuming their final destination.

### A putative anti-apoptotic role for *Pax7 *in postnatal superior collicular development

As earlier quantification did not detect any numerical changes in the *Pax7 *cellular profile in *Pax7*^+/- ^mice, it became apparent that these cells had either altered their differentiation profile or had regressed within the first postnatal week. We assessed *GFAP *expression postnatally and as there was no change in the cellular distribution of GFAP^+ ^cells in *Pax7 *mutant mice we excluded cell fate switching or transdifferentiation to the astrocytic lineage as a causative factor for the reduced number of Pax7^+ ^cells dorsally. We were, however, unable to detect increased apoptosis within the superior colliculus using activated *caspase 3*, cresyl violet or Hoechst staining. A more detailed examination commencing from birth would be required to determine the exact time interval when perturbation occurs in order to conclusively demonstrate apoptotic mechanisms. Apoptotic labelling methods such as TUNEL or caspase 3, concomitant with Pax7 immunolabelling, would be required to address this issue, however rapid neuronal degeneration coupled with a progressive rostrocaudal maturation to the tissue which is likely to result in a punctuated apoptotic phenomenon, will likely impede the quantitative nature of the analysis required to differentiate between normal (wildtype) and increased (mutant) apoptosis. However, the loss of neurons evidenced in *Pax7 *mutant mice indicates a loss of cells, rather than simply extinction of *Pax7 *expression.

**Figure 9:**
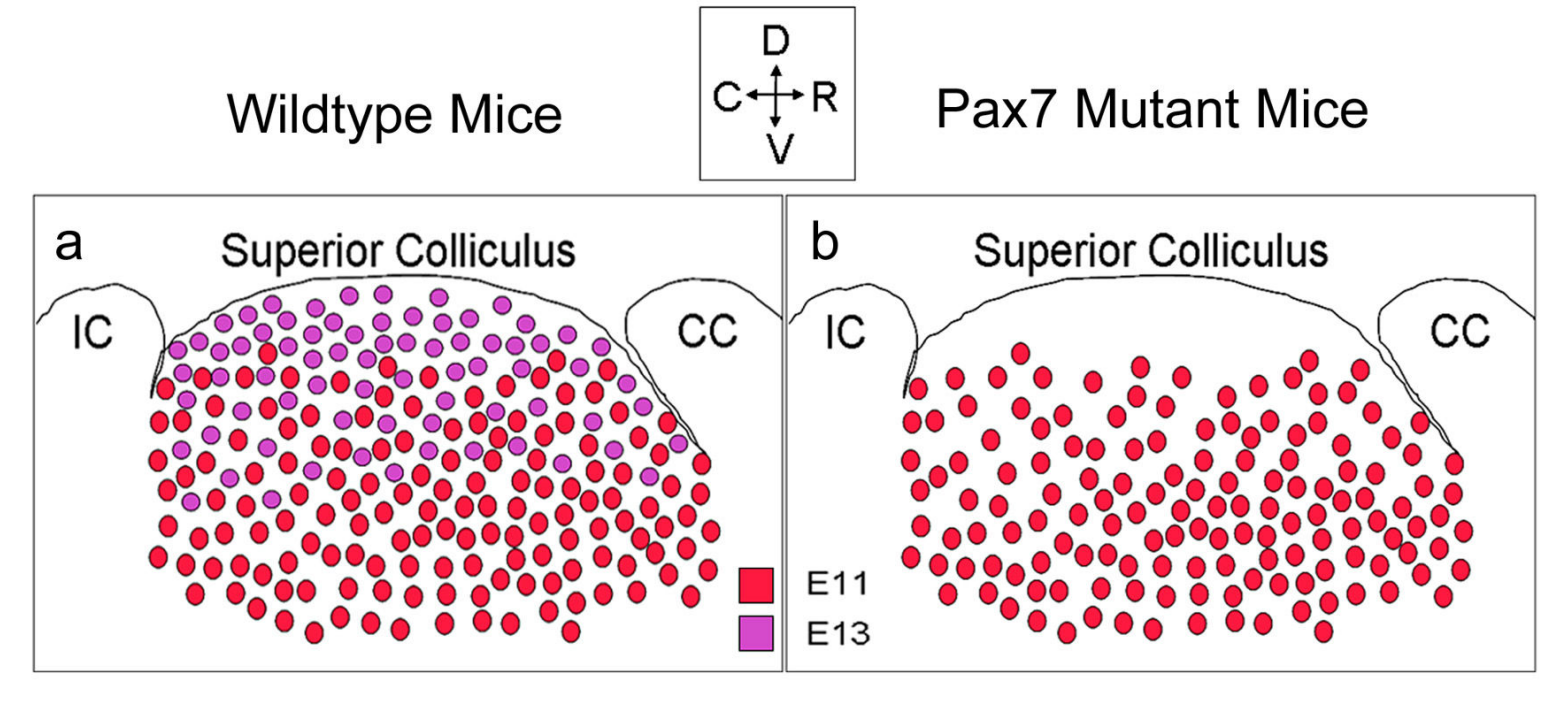
**A diagram detailing differential placement of separately generated populations of Pax7+ cells at P5**. A diagram of Pax7^+ ^cells in the superior colliculus of wildtype (a) and *Pax7 *mutant (b) mice at P5. Cells generated at E11 (red) and E13 (purple) show overlapping (medial) and unique (ventral [E11]/dorsal [E13]) expression patterns (a). We propose that cells generated at E13 show reduced capacity for long term maintenance in *Pax7 *mutant mice (b), generating a phenotype whereby a superficial region is absent of Pax7^+ ^cells, and the region immediately ventral to this exhibits a reduction in the number of Pax7^+ ^cells (refer Fig 2e). Abbrev. *CC*, cerebral cortex; *IC*, inferior colliculus.

Relating phenotype to the specification of cells during superior collicular development [14] suggests that the second population of neurons, generated at E13, may be less capable of long-term survival than their earlier-produced counterparts, and as such are sensitive to gene dosage (Fig 9). Whilst the differential placement of late-born neurons identified in previous studies [14,35] coincides spatially with the perturbed neuronal profile of *Pax7 *mutant mice further experimentation is required to distinguish a link between the generation of subpopulations of cells and their relationship with *Pax7 *and postnatal cytoarchitecture. Furthermore, differential sensitivity to neuron death has been suggested for different functional populations of neurons within the superior colliculus [36]. Anti-apoptotic roles for *Pax *genes are well-documented to-date [37-41] and, importantly, the ciliary neurotrophic factor receptor has recently been demonstrated as a downstream target of Pax7 within the mouse embryo [42].

### Apparent neuronal recovery at P18.5

We have demonstrated altered Pax7^+ ^and neuronal profiles in *Pax7 *mutant mice at P5, however at P18.5 we can no longer detect these variations between *Pax7 *mutant and wildtype mice. Whilst it is feasible that the similar number of Pax7^+ ^cells in *Pax7*^+/- ^mice relative to wildtype at P18.5 occurs because Pax7^+ ^cells in the adult do not normally include the subpopulation of cells which are missing at P5 in *Pax7 *mutant mice, the apparent recovery of the neuronal profile by P18.5 has proven enigmatic. We have drawn from established studies of CNS and superior collicular development to address this issue.

It is clear that early postnatal stages of superior collicular development are tumultuous. The colliculus becomes invaded by retinal afferents just prior to birth and this continues within the first postnatal week, with formation of collaterals giving rise to axonal arborisation and commencement of synaptogenesis [15,43,44]. During this process, a dramatic transformation occurs in which almost half of all fibre bundles are removed from the upper half of the *stratum superficiale *(the region including the perturbation where Pax7^+ ^cells are essentially absent) [15,44]. This process coincides with naturally occurring neuron death in the superior colliculus [36,45], indicating neuronal formation in excess of adult requirements. Excessive neuron formation is a recurrent theme in CNS development [22,46,47]. Therefore it is likely that excessive neuron formation masks the loss of the more susceptible, Pax7-deficient neurons, with sufficient cells remaining to satisfy functional requirements. Subsequently, displacement of cells due to fibre invasion and the ensuring maturation of the tissue could possibly account for the apparent phenotypic equivalence of NeuN^+ ^cells noted at P18.5. It should be noted that functional studies have not been performed on *Pax7 *mutant mice to-date to assess visual acuity.

### Further studies

A possible cause for the loss of superficial neurons in early postnatal stages could be altered circuitry. Neurons of the superficial superior colliculus communicate with the deeper collicular regions through superficial neuronal axons [48,49], or via dendrites from neurons of the deep layers [50] which in turn contain a variety of efferent projections from regions such as the cortex, retina, zona incerta (region surrounding subthalamus) [32,51-53] and indirectly from the subthalamus via the substantia nigra pars reticulata or enteropeduncular nucleus [54-56]. Taking this into consideration, an interesting finding from this research is the persistent *Pax7*^+^*/Pax3*^- ^expression profile of the subthalamus throughout all timepoints examined. Providing *Pax3 *is not expressed in this region at an earlier developmental stage than examined in this study, the subthalamus may represent a novel opportunity to further dissect the functional repertoire of *Pax7 *from that of *Pax3*. Additional work would therefore be required to characterize the subthalamus of *Pax7 *mutant mice to address this issue, as this important brain region is a target of deep brain stimulation to treat disorders such as Parkinson's Disease and epilepsy [56,57].

Further studies on the role of *Pax7 *in superior collicular development would benefit from investigation into the axonal projection and circuity of Pax7^+ ^cells, which may be elaborated by the generation of *Pax7/tau*-labelled X *Pax7 *mutant mice. Cell lineage tracing experiments in *Pax7 *mutant mice would be required to trace the progression of cells generated at E11 and E13 to conclusively resolve the questions related to the early postnatal perturbation and the apparent neuronal recovery in the adult. Moreover, functional studies assessing the ability of *Pax7 *mutant mice to evoke a co-ordinated response to stimuli will be required to assess the integrity of the mature superior colliculus.

## Conclusion

In summary *Pax7*, while not required for neuronogenesis or neuronal differentiation at early stages of superior collicular development, is absolutely requisite in a dosage-dependent manner for long term maintenance of a subpopulation of dorsal mesencephalic neurons. This characteristic may well impart prophylactic properties to stem cells utilised in future replacement therapies to enhance treatment of neurodegenerative diseases of the midbrain. Furthermore, comparative expression analyses indicate a functionally divergent role for *Pax7 *and *Pax3 *during neuronal differentiation within the superior colliculus.

## Methods

### Mouse tissue

The generation and genotyping of *Pax7 *mutant mice has been described previously [10]. Principles of laboratory animal care (NIH publication No. 86-23, revised 1985) were followed, and animal experimental procedures conformed to National Health and Medical Research Council of Australia guidelines, with approval by the Animal Ethics Committee of Edith Cowan University. The production of transgenic animals at the Max Planck Institute for Biophysical Chemistry was performed with the approval of LAVES (Landesamt für Verbraucherschutz und Lebensmittelsicherheit) in Oldenburg.

Whole embryos (E12.5) or embryonic brains (>E15.5) from mutant and wildtype littermates were isolated and postfixed in 4% paraformaldehyde. Postnatal mice were deeply anaesthetised with Avertin and intracardially perfused with fixative. Brains were isolated and postfixed with 4% paraformaldehyde. Tissue was cryoprotected in 20% or 30% sucrose/PBS prior to sectioning. Embryos at E12.5 were sectioned whole. For all other stages, brain hemispheres were sectioned in the sagittal (left) or coronal (right) planes at 10–20 μm, and slides were stored at -80°C until required. A minimum of three animals per group (+/+; +/-; -/-) per timepoint were analysed (with the exception of Pax7^-/- ^animals at E18.5, where n = 2).

### Immunohistochemistry

Whole embryo tissue from E12.5 mice was subjected to antigen retrieval by microwave heating in sodium citrate buffer pH6.0 prior to processing. All tissue sections were treated with 0.2% Triton-X100/PBS (10 min), 1.5% H_2_O_2_/PBS (2–3 × 10 min), blocked with 10% fetal calf serum/PBS (30 min) and incubated overnight at 4°C with primary antibodies; Pax7 (1:20 or 1:10 (E12.5), mouse, monoclonal, DSHB, Iowa City, IA, USA); Pax3 (1:100 or 1:50 (E12.5), mouse, monoclonal, DSHB, Iowa City, IA, USA); Pax6 (1:100, mouse, monoclonal, DSHB, Iowa City, IA, USA); En-1 (1:50, mouse, monoclonal, DSHB, Iowa City, IA, USA); ephrin-A2 (1:500, rabbit, polyclonal, Santa Cruz Biotechnology, Santa Cruz, CA, USA); NeuN (1:100, mouse, monoclonal, Chemicon/Millipore, Billerica, MA, USA). Sections were then incubated with biotinylated anti-mouse/anti-rabbit IgG (1:3, Dako, Sydney, NSW, Australia) followed by streptavidin/HRP complex (1:3, Dako, Sydney, NSW, Australia) (20 mins each at room temperature). Visualization with diaminobenzidine (3% in substrate buffer, Dako, Sydney, NSW, Australia) preceeded processing through an ethanol series/xylene, and mounting in DePex. Control slides without primary antibodies were immunonegative.

### Immunohistochemical quantification and analysis

Images were captured with a Leica DC300 camera attached to an Olympus BX41 microscope and analysed using Optimas 6.5 Digital Image Analysis software (Media Cybernetics, Bethesda, MD, USA). For cellular quantification at E15.5 and P18.5 images were captured within Optimas (100× magnification) and Pax7^+ ^cells were marked and counted. For optical density and cell distribution measurements at P5, the microscope was equilibrated prior to analysis, and microscope and image software settings were standardised. Measurements of Pax7 immunostaining were obtained on a frame-by-frame basis (400× magnification), and encompassed the entire Pax7^+ ^portion of the superior colliculus, producing a serial reconstruction of the region (Coggeshall and Lekan 1996). Each individual cell measurement was topographically replotted within Excel and values were normalised against immunonegative adjacent tissue. Results were subsequently analysed producing maps of mean cellular distribution, or graphed as either mean cellular protein level or mean cell number at each axis point. Standard errors are shown.

### Immunofluorescence

Tissue sections were blocked with 3% normal goat serum (NGS)/0.2% Triton-X100/TBS (TX-TBS) for one hour at room temperature and incubated overnight at 4°C with primary antibody diluted in 1% NGS/TX-TBS. Primary antibodies included Pax7 (1:10); Pax3 (1:50); activated-Caspase 3 (1:100, rabbit, polyclonal, Promega, Sydney, NSW, Australia); alpha-internexin (1:150, rabbit, polyclonal, Chemicon/Millipore, Billerica, MA, USA); βIII tubulin (TuJ1)(1:500, rabbit, polyclonal, Abcam, Cambridge, MA, USA); Doublecortin (1:500, rabbit, polyclonal, Abcam, Cambridge, MA, USA); GFAP (1:1000, rabbit, polyclonal, Chemicon/Millipore, Billerica, MA, USA); Ki67 (1:150, rabbit, polyclonal, Abcam, Cambridge, MA, USA). Primary antibodies were visualised with conjugated anti-rabbit IgG-AlexaFluor488 (1:500; Molecular Probes-Invitrogen, Melbourne, VIC, Australia), or biotinylated goat anti-mouse IgG (1:500; Abcam, Cambridge, MA, USA), incubated overnight followed by streptavidin-AlexaFluor546 (1:500; Molecular Probes-Invitrogen, Melbourne, VIC, Australia), incubated for 2 hours at room temperature. Slides were counterstained with Hoechst (1/5000; Sigma Aldrich, Sydney NSW, Australia) and mounted in Fluorsave (Calbiochem, La Jolla CA, USA). Controls with primary antibodies omitted were immunonegative. Fluorescent micrographs and the confocal z-stack image (see Additional file 1) were obtained using Confocal Assistant software (Version 4.02) and the Biorad MRC 1000/1024 Confocal Microscope or Olympus BX41 microscope equipped with an Olympus DP71 camera.

### Statistical analysis (Table 1)

To assess variation between wildtype and mutant mice at E15.5, P5 and P18.5, we compared the number of Pax7^+ ^cells in wildtype mice with those in *Pax7*^+/- ^mice using paired *t *test (two-tailed). For determination of graded expression at P5, mean values at three points across each axis (rostral, mid and caudal, or ventral, mid and dorsal) were calculated by averaging cell numbers in two frames at each axis position. Graded expression across each axis was assessed by analysis of significant variation between axis points using the paired *t *test (two-tailed): rostral vs mid (R-M), mid vs caudal (M-C), rostral vs caudal (R-C) or ventral vs mid (V-M), mid vs dorsal (M-D), ventral vs dorsal (V-D). Corresponding significance values are reported in Table 1. All statistical analyses were conducted utilising SPSS Version 13.0 Statistical Software, with a 95% confidence interval.

## Authors' contributions

AM initially generated and currently maintains the *Pax7 *mutant mouse line, and critically reviewed the manuscript. AZ performed handling, genotyping, perfusion and initial processing of *Pax7 *mutant mice, and revision of the manuscript. JT participated in the design of the study and further processed the tissue, performing all subsequent experimentation and analysis, including drafting of the manuscript. MZ participated in the design and coordination of the study and editing of the manuscript. All authors read and approved the final manuscript.

## Supplementary Material

Additional file 1 **Confocal z-section micrograph detailing *βIII tubulin *and *Pax7 *expression in the superior colliculus at E15.5**. βIII tubulin (green) and Pax7 (red) immunostaining within the mouse superior colliculus at E15.5 (wildtype). Note the more dorsal positioning of the Pax7^+ ^cells, and the rostrocaudally aligned axons penetrating through the rostral superior colliculus (left) towards the mid region, with superficial axons close to the pial surface penetrating more caudally (right). The neurons of the *stratum profundum *are visible in the ventral regions, superior to the ventricle in the bottom right of the image. Magnification; 100×. Click here for file
