# Supplementary figures and images for: Pax7 is requisite for maintenance of a subpopulation of superior collicular neurons and shows a diverging expression pattern to Pax3 during superior collicular development
Source: BMC Dev Biol. 2008 May 30;8:62. doi: 10.1186/1471-213X-8-62 (PMC2430198; doi:10.1186/1471-213X-8-62)

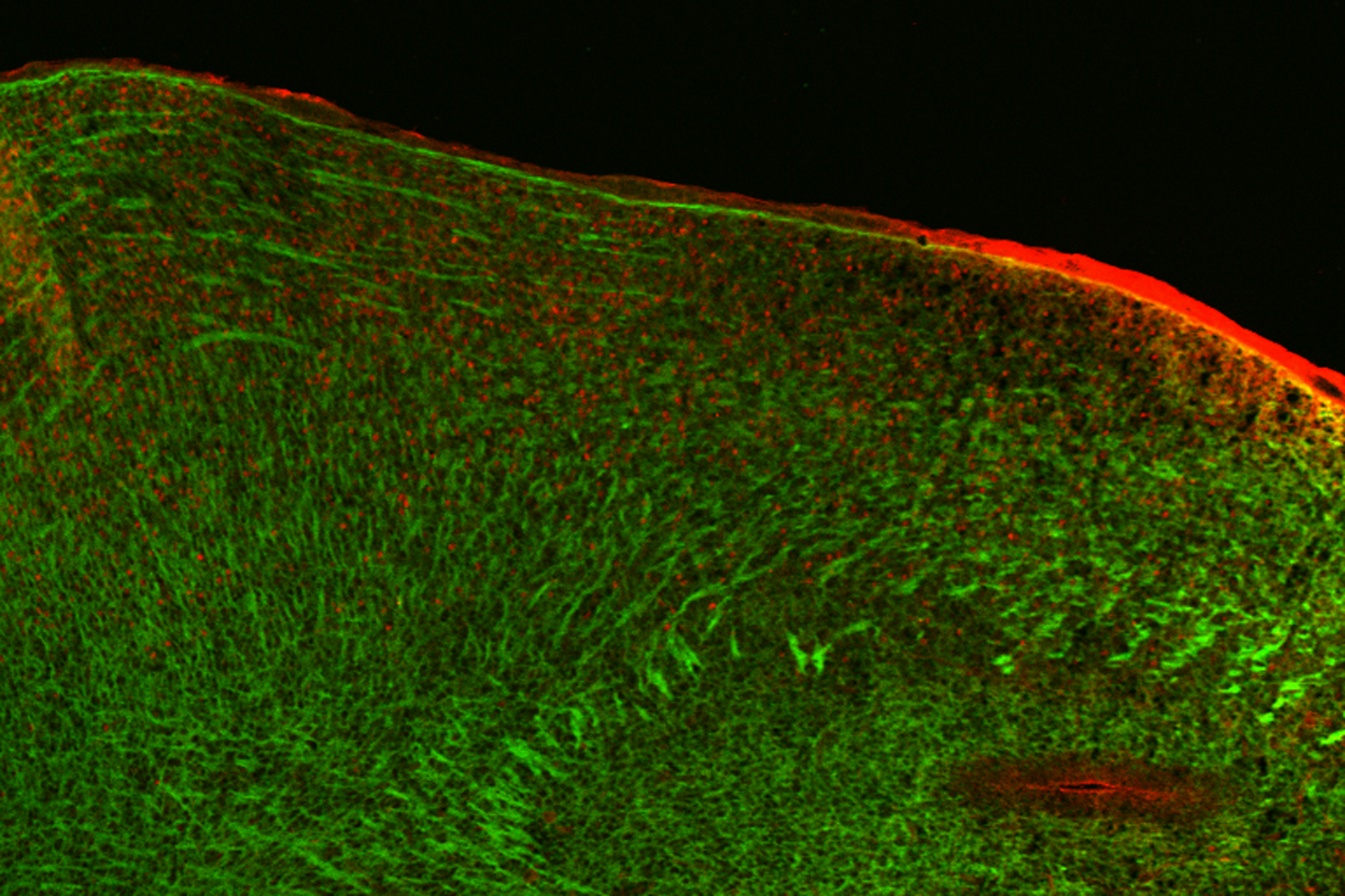

Supplement: Additional file 1 — Confocal z-section micrograph detailing βIII tubulin and Pax7 expression in the superior colliculus at E15.5. βIII tubulin (green) and Pax7 (red) immunostaining within the mouse superior colliculus at E15.5 (wildtype). Note the more dorsal positioning of the Pax7+ cells, and the rostrocaudally aligned axons penetrating through the rostral superior colliculus (left) towards the mid region, with superficial axons close to the pial surface penetrating more caudally (right). The neurons of the stratum profundum are visible in the ventral regions, superior to the ventricle in the bottom right of the image. Magnification; 100×. [file 1471-213X-8-62-S1.tiff]
